# Neuroprotective Effect of Tauroursodeoxycholic Acid (TUDCA) on *In Vitro* and *In Vivo* Models of Retinal Disorders: A Systematic Review

**DOI:** 10.2174/1570159X21666230907152207

**Published:** 2023-09-08

**Authors:** Jiaxian Li, Ziyang Huang, Yu Jin, Lina Liang, Yamin Li, Kai Xu, Wei Zhou, Xiaoyu Li

**Affiliations:** 1 Department of Eye Function Laboratory, Eye Hospital, China Academy of Chinese Medical Sciences, Beijing 100040, China

**Keywords:** Tauroursodeoxycholic acid, TUDCA, neuroprotection, retinal disorder, retinal degeneration, retinitis pigmentosa, diabetic retinopathy, traditional Chinese medicine

## Abstract

**Background:**

Tauroursodeoxycholic acid (TUDCA) is a naturally produced hydrophilic bile acid that has been used for centuries in Chinese medicine. Numerous recent *in vitro* and *in vivo* studies have shown that TUDCA has neuroprotective action in various models of retinal disorders.

**Objectives:**

To systematically review the scientific literature and provide a comprehensive summary on the neuroprotective action and the mechanisms involved in the cytoprotective effects of TUDCA.

**Methods:**

A systematic review was conducted in accordance with the PRISMA (The Preferred Reporting Items for Systematic Reviews and Meta-Analyses) guidelines. Systematic literature search of United States National Library of Medicine (PubMed), Web of Science, Embase, Scopus and Cochrane Library was performed, which covered all original articles published up to July 2022. The terms, “TUDCA” in combination with “retina”, “retinal protection”, “neuroprotection” were searched. Possible biases were identified with the adopted SYRCLE’s tool.

**Results:**

Of the 423 initially gathered studies, 24 articles met inclusion/exclusion criteria for full-text review. Six of them were *in vitro* experiments, 17 studies reported *in vivo* data and one study described both *in vitro* and *in vivo* data. The results revealed the effect of TUDCA on different retinal diseases, such as retinitis pigmentosa (RP), diabetic retinopathy (DR), retinal degeneration (RD), retinal ganglion cell (RGC) injury, Leber’s hereditary optic neuropathy (LHON), choroidal neovascularization (CNV), and retinal detachment (RDT). The quality scores of the *in vivo* studies were ranged from 5 to 7 points (total 10 points), according to SYRCLE’s risk of bias tool. Both *in vitro* and *in vivo* data suggested that TUDCA could effectively delay degeneration and apoptosis of retinal neurons, preserve retinal structure and function, and its mechanism of actions might be related with inhibiting apoptosis, decreasing inflammation, attenuating oxidative stress, suppressing endoplasmic reticulum (ER) stress, and reducing angiogenesis.

**Conclusion:**

This systematic review demonstrated that TUDCA has neuroprotective effect on *in vivo* and *in vitro* models of retinal disorders, reinforcing the currently available evidence that TUDCA could be a promising therapeutic agent in retinal diseases treatment. However, well designed clinical trials are necessary to appraise the efficacy of TUDCA in clinical setting.

## 1 INTRODUCTION

**Fig. (1):**
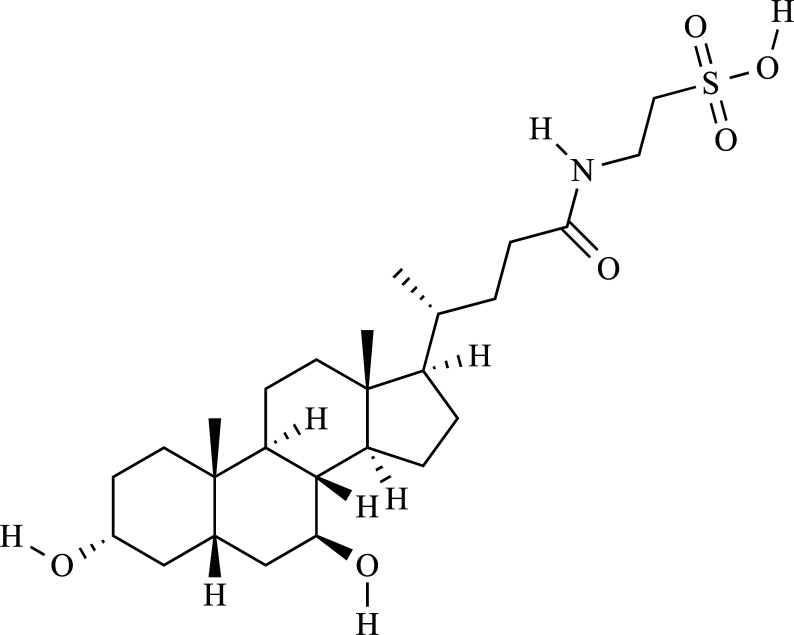
Chemical structure of tauroursodeoxycholic acid.

For centuries, traditional Chinese medicine (TCM) has valued the use of animal bile for pharmacological and clinical applications [1]. Bile acids are produced in the liver and excreted into the intestine, participating in the emulsification, absorption, and digestion of lipids. As steroid hormones, they regulate various metabolic processes, such as liver glucose metabolism and hepatocyte survival [2, 3]. The beneficial effects have been noted in ancient TCM literature, which include detoxifying the liver, dissolving kidney stones and gallstones, stopping convulsions, and improving vision [4]. The unique chemical composition contributes to their anti-inflammatory, anti-oxidant, anti-microbial, anti-convulsant, and anti-hepatotoxic effects, which explains their historical application in TCM [1, 5-8]. Currently, forty-four different animal bile products isolated from vertebrates and invertebrates have been used to treat liver, skin, and biliary system ailments, especially bile and gallbladder collected from black and brown bears [1, 6]. The hydrophobicity of bile acids decreases with increased hydroxyl groups. Hydrophilic bile acids are considered excellent ingredients for the treatment of neurodegenerative diseases. TUDCA, a taurine conjugate of ursodeoxycholic acid (UDCA), is among the most hydrophilic bile acids [9, 10]. The chemical structure of TUDCA is C_26_H_45_NO_6_S (Fig. **1**) and its molecular weight is 499.7 (https://pubchem.ncbi.nlm.nih.gov/, accessed September 22, 2022).

The main pathway of its biosynthesis includes that microsomal cholesterol 7 α-hydroxylase converts cholesterol to 7 α-hydroxycholesterol. Then, under the catalysis of 3β-hydroxy-Δ5-C27-steroid dehydroxylase, 7α-hydroxycholesterol turns into 7α-hydroxy-4-cholestene-3-one. It is followed by a series of reactions, including the cleavage of steroid side chains catalyzed by mitochondrial sterol 27-hydroxylase to form chenodeoxycholic acid (CDCA). Alternatively, cholesterol might be converted to 27-hydroxycholesterol by sterol 27-hydroxylase, then hydroxylated to 3β-7α-dihydroxy-5-cholestenoic acid by oxysterol 7α- hydroxylase, which is finally converted to CDCA by sterol 27-hydroxylase. CDCA and its conjugates are secreted with bile to the large intestine, where the gut microbiota converts them into UDCA. Finally, UDCA returns to the liver through enterohepatic circulation and binds with taurine to form TUDCA [11-13].

Numerous studies have reported neuroprotective effects of TUDCA in various neurodegenerative disease models, which can cross the blood-brain barrier and exert effects in the brain [14-18], including Parkinson's disease (PD) [19-21], Alzheimer's disease (AD) [22-26], and Huntington’s disease (HD) [27]. TUDCA alleviated mitochondrial dysfunction which was characteristic of PD [27]. In the AD mice model, TUDCA inhibited amyloid precursor protein processing and amyloid-β deposition [24], and significantly attenuated Aβ deposition in the brain after amyloid pathology [26]. c-Jun N-terminal kinase (JNK) played a central role in the death of dopaminergic neurons, reactive oxygen species (ROS) production, and activation of Akt prosurvival pathway, which involved the death promoting factors bad phosphorylation and the activation of nuclear factor kappa-B (NF-κB). TUDCA exerted neuroprotective effects by modulating the activity of JNK in a mouse model of Parkinson's disease [20]. TUDCA improved the locomotor and sensorimotor abilities of the HD model, while reducing striatal cell apoptosis and intracellular Huntington protein inclusion bodies [16]. In brains, studies have shown that TUDCA binds to G protein-coupled bile acid receptor 1/Takeda G protein-coupled receptor 5, resulting in increased levels of intracellular cyclic adenosine monophosphate in microglia, which induce anti-inflammatory markers while reducing pro-inflammatory markers [28]. In addition, TUDCA counteracted amyloid beta-peptide-induced neuronal apoptosis by binding to mineralocorticoid receptors in primary neurons [29]. Recent research also proved that TUDCA promoted the survival, proliferation, and transformation of stem cells [30-32]. Also, its beneficial effects involved cardiovascular disease [33-35], diabetes [36-38], obesity and cancer [39, 40].

With the in-depth study of retinal neurons, TUDCA has shown protective effects in several retinal disorder models, such as photoreceptor degeneration, retinal ganglion cell (RGC) injury, diabetes retinopathy (DR), laser-induced choroidal neovascularization (CNV) [29]. The retina is a kind of light-sensitive tissue that lines the inner surface of the eye and is responsible for the first step in visual processing. The complexity of the structure and function of the retina makes it susceptible to changes by any sort of pathological damage [41]. As first-order neuron that reconverts light energy into visual signals, photoreceptors are responsible for capturing different wavelengths of light over a wide range of brightness. Thus, healthy photoreceptors are critical for vision. The loss of photoreceptors rapidly leads to visual dysfunction and, ultimately, retinal remodeling in the advanced stages of the disease due to the loss of afferent secondary and tertiary retinal neuron signals [4, 42-45]. These pathological changes are common in retinitis pigmentosa (RP), age-related macular degeneration (AMD), and other types of retinal degeneration (RD).

Besides photoreceptors, vision also depends on the condition of retinal ganglion cells (RGCs), which provide the final circuit between retinal processing and higher-order visual processing in the midbrain and cortex. If RGCs are damaged, visual information cannot be transmitted to the midbrain for processing and interpretation [4]. Therefore, its final result is permanent blindness which could happen in glaucoma, anterior ischemic optic neuropathy, traumatic optic neuropathy, *etc*.

DR is another leading cause of vision loss. It has been described as a fundus microvascular disease that clinically manifests as structural abnormalities of the retinal vasculature. However, numerous studies have shown that DR still affected retinal neurons, including photoreceptors and RGC abnormalities [4, 46]. Almost all of the above-mentioned retinal disorders are progressive and lead to visual loss and eventual blindness. Consequently, it is urgent to study an effective neuroprotective agent to delay the degeneration and death of retinal cells.

Excitedly, as mentioned ahead, TUDCA is a powerful neuroprotective agent with the potential to have extensive protective effects on the retina. Its advantage is that they might be administered orally, subcutaneously, and intravenously. In most cases, they presented rather low toxicity to the organism [13]. These favorable characteristics also explain why it has been studied as a potential protective agent widely used in many diseases.

Our study aimed to evaluate reports on the neuroprotective effect of TUDCA on *in vitro* and *in vivo* models of retinal disorders through a systematic review of the literature.

## 2 MATERIALS AND METHODS

Our systematic review was designed according to The Preferred Reporting Items for Systematic Reviews and Meta-Analyses (PRISMA) [47] and PRISMA Extension for Chinese Herbal Medicines 2020 (PRISMA-CHM, 2020) [48]. In the systematic review, all of the retrieved studies were related to exploring the effects and the mechanism of action of TUDCA on *in vitro* and *in vivo* models of retinal disorders. Two independent investigators were involved at every stage of the systematic review (studies search and selection, data extraction, and risk of bias assessment).

### 2.1 Searching Strategy

Searches were conducted in PubMed, Web of Science, Embase, Scopus, and Cochrane Library. All original articles published up to July 2022 were searched in parallel by two independent authors. A language restriction was considered for the selection, with only articles published in English being analyzed. A combination of medical subject headings (MeSH) and free text terms were used to identify the disorders and interventions as follows:

i) Tauroursodeoxycholic acid OR TUDCA OR tauroursodeoxycholate OR chenyl taurine sodium OR tauroursodeoxycholate sodium salt

AND

ii) Retina OR retinal protection OR neuroprotection.

The resulting reference lists were manually examined to identify any potential studies that were not captured by the electronic searches. All the articles from these searches were exported to EndNote X8 with duplicate records deleted, as well as articles that were not part of *in vivo* and *in vitro* studies. Articles were firstly screened by reading titles and abstracts, and irrelevant articles or without available full text were excluded. The remaining articles were then screened by reading the full text.

### 2.2 Inclusion and Exclusion Criteria

The inclusion criteria were: 1) *In vitro* or *in vivo* studies; 2) Studies that focused on the effects and action mechanisms of TUDCA on retinal disorders; 3) Independent and full-text accessible original data.

The following exclusion criteria were considered: 1) Studies focused on bear bile extracts other than TUDCA; 2) Studies that analyzed the TUDCA action along with other compounds without an isolated group for TUDCA; 3) Absence of control group (the control group had to be comparable to the group supplemented with TUDCA).

### 2.3 Data Extraction and Management

Two investigators performed data collection independently based on the inclusion and exclusion criteria using an Excel sheet. Discrepancies were assessed by consensus, and when they were not initially reached, the third reviewer was consulted. The following information was extracted from each study: 1) For *in vitro* studies: study title, author, year of publication, disease, type of cells and their origin, the concentration of TUDCA, laboratory techniques, and major findings/mechanism of actions. 2) For *in vivo* studies: study title, author, year of publication, disease, animal’s species, sex and age, type of animal model, number of animals per group (and number of animals in total if specified), routes of administration, the dose of TUDCA, frequency, and duration, follow up, laboratory techniques, tissues studied and major findings.

### 2.4 Quality Assessment

The quality of *in vivo* studies was assessed using a checklist of the Systematic Review Centre for Laboratory animal Experimentation (SYRCLE) based on the Cochrane Collaboration RoB Tool [49]. It consists of ten items within six main domains, namely selection bias, performance bias, detection bias, attrition bias, reporting bias, and other sources of bias. The answer for the judgement of bias was either “YES” to indicate a low risk of bias, “NO” to indicate a high risk of bias, or “NC” to indicate an uncertain level of bias because of insufficient information. For each item, one point was given for “YES”.

## 3 RESULTS

### 3.1 Study Inclusion

A total of 423 articles were extracted from the original literature, of which 102 articles appeared in PubMed, 183 in Embase, 52 in Web of Science, 86 in Scopus and nothing was found in the Cochrane Library. Next, search filters were implemented, which excluded 302 articles (218 duplicates, 61 reviews, and 23 conference abstracts). By reading the titles and abstracts, 36 articles not related to TUDCA and 51 articles not related to retinal diseases were excluded. Thus, 34 articles were read in their full-text. After analyzing these articles, 10 articles failed for at least 1 criterion and were excluded (other types of research, other bear bile extracts, absence of control group, no full-text available). There were 24 articles at the end of the analysis [50-73]. They contained *in vivo* (17 studies), *in vitro* (6 studies) experiments, and one study was conducted both. The process and results were summarized in Fig. (**2**). Further, we found that studies analyzed TUDCA actions on different retinal injuries: RP (8 studies), DR (6 studies), RD (4 studies), RGCs injury (2 studies), Retinal pigment epithelium damage (2 studies), Leber’s hereditary optic neuropathy (LHON, 1 study), CNV (1 study) and retinal detachment (RDT, 1 study).

### 3.2 In Vitro

#### 3.2.1 Study Characteristics

*In vitro* studies were performed on seven different cells and retinal explants (RE), three of these were performed on RE from rats (2 from SD rats and 1 from Wistar rats). Two studies were performed on ARPE-19 (retinal pigment epithelial cell line) cells. The remaining studies covered WERI-Rb-1 human cone cells, retinal neural cells from Wistar rats, human retinal microvascular endothelial cells (HRMECs), primary human retinal pigment epithelium (hRPE), photoreceptor outer segment (POS) from porcine eyes. One study was conducted simultaneously on 3 cells and explants, and the other one was conducted on 2 species.

The retinal damage models were induced by exposure to different substances. In terms of RD, 100 ug/ml glucose-advanced glycation end products-bovine serum albumin (glucose-AGE-BS, 1 study) and 12, 20 mg/mL albumin (1 study) were used; DR was mimicked by culturing in high glucose conditions (25-45 mM or 4.5 g/L glucose, 3 studies); as for retinal pigment epithelium (RPE) damage, 100-750 µM hydrogen peroxide (H_2_O_2_) exposure (2 studies, in one of these studies simultaneously 1 µM thapsigargin exposure) were applied.

The studied TUDCA concentrations were in a range from 0.2 µM to 300 µM (except that one study used 10 ng/ml). Among all studies, the concentration of 100 µM (4 out of 7 studies) was frequently used. Regarding about treated duration of TUDCA, the minimum duration of *in vitro* studies was 2 hours and the maximum was 7 days.

The laboratory techniques frequently used *in vitro* studies were Western blot (WB, 5 studies), terminal-deoxynucleotidyl transferase-mediated nick end labeling (TUNEL staining, 5 studies), immunohistochemistry (IHC), and immuno-cytochemistry (ICC, 4 studies), fluorescence probe (2',7'-dichlorodihydrofluorescein diacetate (H_2_DCFDA), 3 studies), enzyme-linked immunosorbent assay (ELISA, 2 studies), *etc*. *In vitro* studies on the TUDCA effect in cells and tissues of retinal disorders were summarized in Table **1** [50-56].

#### 3.2.2 The Effect of TUDCA on Cells Activity and Apoptosis

Bikbova *et al*. [50] demonstrated that TUDCA not only reduced the expressions of JNK but also caspase-9 in the ganglion cell layer (GCL), thereby decreasing the number of apoptotic cells (measured with IHC). Daruich *et al*. [51] showed that pre-treatment of cone-like cells with TUDCA could protect cells from albumin toxicity. On RE, TUDCA reduced apoptosis, necroptosis, and microglia activation(measured with IHC, WB). RNA-seq showed that it regulated 463 genes, mainly involved in iron control, cell death, oxidative stress, and cell metabolism. The up-regulated genes were involved in the endoplasmic reticulum (ER) stress pathways and the down-regulated genes were involved in axonal and neuronal development (measured with RNA sequencing). Gaspar *et al*. [52] found that TUDCA decreased cells death in cultured retinal neural cells induced by exposure to elevated glucose concentration and prevented apoptosis-inducing factor (AIF) release and mito-nuclear translocation in mitochondria (measured with TUNEL staining, ICC). Toshiyuki *et al*. [53] demonstrated that increased expressions of phosphorylated c-Jun (p-c-Jun) and phosphorylated JNk (p-JNK) were associated with neuronal cell death in diabetic rat retinas and retinas exposed to high glucose. The neuroprotective effect of TUDCA was correlated with the suppression of p-c-Jun and expression of p-JNK (measured with WB). Alhasani *et al*. [56] revealed that TUDCA treatment increased the cell activity of ARPE-19 cells that were exposed to H_2_O_2_ and decreased cell apoptosis by inhibiting the expression of caspase and the activity of caspase-3,7 (measured with TUNEL staining, caspase assay). Likewise, TUDCA suppressed thapsigargin-induced ER stress and cell apoptosis by decreasing the expression of CCAAT-enhancer-binding protein homologous protein (CHOP) and spliced X-box binding protein-1 (XBP1, measured with qRT-PCR).

#### 3.2.3 The Effect of TUDCA on ROS Production/oxidative Stress

The antioxidant properties of TUDCA might explain its cytoprotection. Gaspar *et al*. [52] demonstrated that biomarkers of oxidative stress, protein carbonyl groups, and ROS production were markedly decreased after TUDCA treatment as compared to cells exposed to elevated glucose concentration alone (measured with H_2_DCFDA). Alhasani *et al*. [56] revealed that TUDCA improved the antioxidant capacity of RPE cells after H_2_O_2_ treatment by reducing the production of ROS and malondialdehyde (MDA), upregulating the expression of antioxidant genes, and increasing the generation of glutathione (GSH). Furthermore, it was also related to upregulating the expression of nuclear factor erythroid 2-related factor 2 (NRF2) at the mRNA level and increasing the expression and activity of its downstream target antioxidant enzymes (measured with qRT-PCR).

#### 3.2.4 The Effect of TUDCA on Inflammatory Cytokines

Inflammation is critical for the progression of plenty of retinal disorders. Wang *et al*. [54] demonstrated that TUDCA inhibited inflammation in H_2_O_2_-challenged HRMECs by decreasing the expression of proinflammatory including cytokines intercellular adhesion molecule-1 (ICAM-1), nitric oxide synthase (NOS), NF-κB P65 (measured with WB). Alhasani *et al*. [56] showed that TUDCA alleviated H_2_O_2_-induced inflammation in RPE cells by decreasing the expression and secretion of proinflammatory cytokines, Interleukin-1β (IL-1β), Interleukin-6 (IL-6), and Tumor necrosis factor-α (TNF-α), which may help to maintain RPE function and benefit photoreceptor cells (measured with qRT-PCR).

#### 3.2.5 Other Cellular Effects of TUDCA

Murase *et al*. [55] showed that TUDCA enhanced phagocytosis of POS and reversed H_2_O_2_-induced phagocytosis impairment in RPE cells *via* activation of the Mer tyrosine kinase receptor (MerTK) pathway (measured with WB), including MerTK and focal adhesion kinase (FAK), which may contribute to the prevention of photoreceptor degeneration under stress condition. It was worth mentioning that two studies [50, 53] reported that TUDCA exerted a promotion effect of regenerating neurites under high glucose conditions and that effect was correlated with the suppression of p-JNK and p-c-Jun (measured with ICC, WB). Moreover, Wang *et al*. [54] supported the fact that the expression of vascular endothelial growth factor (VEGF) protein induced by high glucose in HRMECs could be decreased by TUDCA, which could ameliorate DR by preventing retinal neovascularization (measured with WB).

### 3.3 In Vivo

#### 3.3.1 Study Characteristics

The studies were conducted on laboratory animals which included Sprague-Dawley (SD) rats (4 studies), Pro 23 His (P23H) rats (3 studies), Brown Norway (BN) rats (2 studies), Rd10 mice (3 studies), C57BL/6 mice (2 studies), B6.C3-rd1 and C57BL-rd1 mice (1 study), Rpgr knockout mice (1 study), Bbs1^M390R/M390R^ mice (1 study), Lrat-/- mice (1 study), BALB/c mice (1 study). Two different kinds of mice were selected in three studies.

Animal models of RP (8 studies) were established by genetic engineering, including P23H mutant rats, rd10 mutant mice, Rpgr knockout mice, and Bbs1 mutant mice. One of these studies used balb/c mice to establish a model of RD induced by light damage (5000 lux for 8 h). Models of RD (2 studies) were induced by different methods, one of which used different strains of rd1 mutant mice (B6.C3 and C57BL). Another study established the model by intraperitoneal injection of N-methyl-N-nitrosourea (MNU) at 60 mg/kg (C57BL/6 mice). Streptozotocin (STZ) was used to establish DR models (3 studies; 30, 50, and 60 mg/kg intraperitoneal administration respectively), two of which were in SD rats and one in C57BL/6J mice. RGCs injury models (2 studies) were established in SD rats by intravitreal injection of N-methyl-D-aspartate (NMDA) 20 mM and optic nerve crush surgery. CNV models (1 study) of BN rats were induced by a 512 nm argon laser. RDT (1 study) was established by transscleral subretinal injection of sodium hyaluronate. Lesions from Lrat-/- mutant mice were used to mimic LHON (1 study).

TUDCA was delivered intraperitoneally (11 studies; doses ranging from 100 to 750 mg/kg), subcutaneously (5 studies; 500 mg/kg), in eye drops (1 study; 100 mM), and in poly D, L-lactic-co-glycolic acid microspheres (PLGA MSs) for intravitreal injection. Among all studies, the dose of 500 mg/kg (15 out of 18 studies) was used most frequently. Regarding about treated duration of TUDCA, the minimum duration in the *in vivo* study was 1 day and the maximum was 60 days.

The frequently used laboratory techniques *in vivo* studies were IHC, immunolabeling and immunofluorescence (13 studies), electroretinogram (ERG, 9 studies), hematoxylin-eosin staining (HE staining, 8 studies), TUNEL staining (6 studies), retinal flat-mounts (6 studies), WB (4 studies), ELISA (3 studies), polymerase chain reaction (PCR, 3 studies) and optical coherence tomography (OCT, 2 studies). *In vivo* studies on the effect of TUDCA in animal models of retinal damage were summarized in Table **2** [57-73].

In the review, the SYRCLE checklist was used to evaluate the risk of bias for 18 included studies. The items evaluated as “YES” were scored one point, and the scores of 10 items were added together for the quality score of each study. The quality scores ranged from 5 to 7 points. There were low risks of bias in baseline characteristics, blinding items of detection bias, attrition, reporting, and other sources. However, only 5 studies mentioned “randomization”, but did not introduce specific methods, and the rest did not report the sequence generation. In addition, for the allocation concealment items in selection bias, the random outcome assessment of detection bias, all the studies were evaluated as “NC”, which mainly resulted in the middle quality of included studies. The bias risk of *in vivo* studies was summarized in Table **3**.

#### 3.3.2 The Effect of TUDCA on Retinal Function

Nine studies provided a functional evaluation of retinas after treatment with TUDCA. Phillips *et al*. [57] demonstrated that scotopic ERG responses were twofold greater in rd10 mice treated with TUDCA, likewise, photopic responses were also twofold larger in TUDCA-treated mice. Fernández-Sánchez *et al*. [58] revealed that the amplitudes of ERG a- and b-waves were enhanced in TUDCA-treated P23H rats under both scotopic and photopic conditions. Brian Oveson *et al*. [59] showed that mean amplitudes of scotopic a- and b-waves, and photopic b-waves increased in mice treated with TUDCA. Retinal function was significantly decreased after light exposure for 7 days, while after treatment of TUDCA, it partially prevented retinal functional damage. Drack *et al*. [60] found that in both Rd10 and Bbs1 mice, amplitudes of scotopic ERG b-waves were enhanced in TUDCA-treated and a-waves were also increased, but this failed to reach statistical significance. Fernández-Sánchez *et al*. [62] revealed that amplitudes of a and b-waves in dark adaptation were increased by 28% and 22% (measured with ERG) respectively in TUDCA-PLGA-MSs-treated P23H rat eyes. Lawson *et al*. [65] demonstrated that B6.C3-rd1 mice treated with TUDCA had preserved photopic b-wave amplitudes (measured with ERG). However, C57BL-rd1 mice exhibited no measurable a-or b-waves. It showed that TUDCA had a better therapeutic effect on B6.C3-rd1 mice. Gómez-Vicente *et al*. [69] revealed that in the models of NMDA - induced retinal injury in rats, the pSTR and nSTR amplitudes of ERG were increased after TUDCA treatment. The amplitudes of scotopic a-waves and b-waves increased by 61% and 66% respectively, and the deleterious effect of NMDA on the scotopic OPs was not obvious. Fu *et al*. [68] demonstrated that early intervention of TUDCA preserved visual function in STZ-mouse models of type I diabetes, which appeared to ameliorate a-waves, b-waves, and oscillatory potential 2(OP2) deficits (measured with ERG). Meanwhile, spatial frequency and contrast sensitivity threshold assessed by the optomotor response (OMR) maintained levels comparable to normal mice. The late intervention of TUDCA showed slight preservation effects compared to early intervention. Tao *et al*. [66] found that in the mice models of photoreceptor degeneration induced by MNU, the scotopic and photopic b-waves amplitudes were increased after TUDCA treatment, as well as the visual acuity and contrast sensitivity. It suggested that TUDCA conferred beneficial effects on the optokinetic performance of mice dealt with MNU (measured with vision-guided optokinetic tests). TUDCA therapy could restrain the spontaneous firing response, enhance the light-induced firing response, and preserve the basic configurations of the ON-OFF signal pathway in degenerative retinas (measured with the MEA system). To conclude, in different retinal injury models, TUDCA treatment increased a- and b-waves amplitudes in both scotopic and photopic adaptation, indicating that this compound had a protective effect on the retinal function.

#### 3.3.3 The Effect of TUDCA on Retinal Morphology

Seven studies observed the protective effects of TUDCA on retinal morphology. Phillips *et al*. [57] demonstrated that the cone outer segments (OS) were visible and the cone opsin labeling appeared as small round structures or punctuate in the region of OS after TUDCA therapy in rd10 mice, and those retinas presented with a continuous line of OS and longer cone OS (paraffin sections were labeled with antic one opsin). Therefore, TUDCA therapy preserved photoreceptor numbers and the morphology of the inner segment (IS) and OS in rd10 mice. Oveson *et al*. [59] showed that in BALB/c mice exposed to intense light for 8 hours, photoreceptors were degenerated due to oxidative damage, and the retinal lesions showed loss of the outer segments of the cone and flattening of the inner segments, while mice treated with TUDCA showed preservation of cone inner and outer segments (labeled by rhodamine-conjugated peanut agglutinin [PNA]). The same protective effect was also observed in the rd10 mice. Drack *et al*. [60] showed that the overall morphology of photoreceptor cells in rd10 mice and bbs1 mice treated with TUDCA was better preserved, and the preservation of outer nuclear layers (ONL) and IS/OS layer was most obvious (measured with HE staining). Tao *et al*. [66] revealed that C57BL/6 mice model of MNU-induced RD, the retinal structure was severely damaged and ONL was lost. While several layers of cell nucleus were retained in ONL with relatively intact inner/outer segments after TUDCA treatment. It suggested that TUDCA treatment could effectively protect the retinal structure (measured with PNA staining and M- and S-cone opsin staining). Wang *et al*. [54] found that in STZ-induced diabetes retinopathy rats, RGCs crossed the inner limiting membrane, with a rough surface, irregular arrangement of inner nuclear layers (INL), and ONL, and enlarged intercellular space. After treatment with TUDCA (500 mg/kg and 250 mg/kg), the cells were regularly arranged, and the morphology of RGCs was improved (measured with HE staining). Two studies have focused not only on photoreceptor morphology but also on its secondary effects on photoreceptor connectivity and the structure of inner retinal cell layers. Fernández-Sánchez *et al*. [58, 62] demonstrated that both intravitreal and systemic treatment of TUDCA in P23H rats could protect the density, structure, and function of photoreceptors, preserve synaptic interactions between photoreceptor cells and secondary neurons, including bipolar and horizontal cells. Presynaptic and postsynaptic structures, as well as synaptic contacts within the outer plexiform layer (OPL) were improved. Furthermore, the number of both rod bipolar and horizontal cell bodies, as well as the density of their synaptic terminals in the OPL, were increased (measured by immunolabeling with specific antibodies). These results suggested that the protective effects of TUDCA on the morphology of retinas extended to other types of retinal cells besides photoreceptor cells.

#### 3.3.4 The Effect of TUDCA on Retinal Cells Apoptosis and Degeneration

Twelve studies demonstrated that TUDCA reduced retinal cell death and degeneration. The thickness of ONL is an essential indication of assessing photoreceptors death, especially the rods. ONL thickness is usually measured by counting the number of nuclei or photoreceptor rows. Fernández-Sánchez *et al*. [58, 62, 64] found that TUDCA treatment effectively preserved the number of photoreceptor rows in P23H rats, especially in central areas of the retina, where the number of photoreceptors was threefold than untreated animals. Meanwhile, TUDCA reduced the number of apoptotic cells (measured with IHC, HE, and TUNEL staining), indicating that its effect was related to anti-apoptosis. In rd10 mice [59], TUDCA-treated mice had thickened ONL at 3 of 6 sites and increased cone density in all four quadrants (measured with HE staining). Similar results were also observed in the rd1 mice [65]. In Rpgr cko mice [63], TUDCA treatment resulted in a greater number of photoreceptors compared with untreated, indicating that the thickness of the ONL was preserved (measured with HE staining). TUNEL assay found that, the number of photoreceptors undergoing cell death was reduced in the treated retina. This phenomenon was possibly related to the fact that TUDCA inhibited the caspase-dependent apoptosis pathway in the retinas of cko mice, as expressions of caspase-3 on both protein and messenger RNA levels were markedly reduced (measured with qRT-PCR). Tao *et al*. [66] found that TUDCA therapy effectively alleviated MNU-induced toxic effects on mice retinas (measured with IHC). A substantial proportion of photoreceptors were retained in treated mice retinas, suggesting that TUDCA could ameliorate RD and slow down the loss of photoreceptors. The real-time quantitative Polymerase Chain Reaction (qRT-PCR) was performed to determine the mRNA levels of apoptotic factors in retinal tissues. The results revealed that TUDCA downregulated the expressions of apoptosis-related factors Caspase-3, calpain-2, and Bax, and upregulated the anti-apoptotic factors, such as B-cell lymphoma 2 (Bcl2). These findings suggested that the anti-apoptotic mechanisms were at least partially involved in TUDCA-mediated protection. Zhang *et al*. [73] showed that TUDCA-treated Lrat^–/–^ mice had a great increased number of cone cells, temporal and central retinal cones are 3 times denser than untreated animals (measured with retinal flat mounts). CHOP, a well-characterized unfolded protein response target gene, is an ER stress marker associated with apoptosis. A substantial reduction of CHOP was observed in the ventral retinas of TUDCA-treated mice. Judged by the activation of Caspase-3, apoptotic signals in ONL were eliminated after being treated with TUDCA (measured with IHC). Moreover, It slowed down cone degeneration and promoted the degradation of cone membrane-associated proteins by enhancing the ER-associated proteins degradation pathways (measured with WB). Mantopoulos *et al*. [72] demonstrated that TUDCA preserved photoreceptors after RDT, reduced the number of TUNEL-positive cells, and prevented the probability of ONL thickness reduction (measured with TUNEL). These effects were associated with the suppression of caspases-3 and -9, as well as carbonyl-protein adducts. It was worth mentioning that TUDCA did not significantly decrease ER stress after RDT. Yang *et al*. [67] indicated that TUDCA protected RGCs from death (measured with HE staining) in diabetic retinas by inhibiting the expressions of caspase-12 and p-c-Jun 1 (measured with immunofluorescence colocalization). The CHOP pathway directly promoted glial reactivity that subsequently contributed to neuron loss and vascular abnormalities in DR. While TUDCA reduced CHOP expressions in diabetic rats. Therefore, TUDCA prevents DR by inhibiting the activation of ER stress pathways (measured with immunofluorescence, WB, and RT-PCR). Other studies [69, 70] reported TUDCA-protected RGCs and axons from injuries induced by NMDA or varies factors.

#### 3.3.5 The Effect of TUDCA on Inflammation

Three of included articles reported the anti-inflammatory effects of TUDCA. Wang *et al*. [54] demonstrated that TUDCA (500 mg/kg and 250 mg/kg) could possibly alleviate inflammation of STZ-induced DR by downregulating NOS, ICAM-1, NF-κB p65 expressions and decreasing the content of NO in rats retinas (measured with IHC), but this effect did not apply in the dose of 750 mg/kg. It might be related to the over-regulation of high-dose TUDCA. Microglial cells acting as the resident immune cells of the central nervous system, including the retina, played the role of primary mediators of inflammation. Noailles *et al*. [61] reported that in P23H rats with a large number of microglia appearing in retinas and subretinal space, TUDCA reduced microglial cells number and prevented activation of them (measured with IHC). Meanwhile, the number and activation of macrophages were also inhibited, and these results supported the anti-inflammatory effect of TUDCA. Zhang *et al*. [63] revealed that TUDCA ameliorated microglia activation by reducing the infiltration of activated microglia into ONL, while inhibiting the formation of inflammasomes by activated microglia and subsequently reducing the expression of the mature inflammatory cytokine IL-1β (measured with cryosectioning and immunostaining).

#### 3.3.6 The Effect of TUDCA on Oxidative Stress

Three studies suggested that TUDCA exerted cytoprotective effects in different models by decreasing oxidative stress. Levels of protein carbonyl adducts, which can indicate ROS levels, can be used as an index of exposure to oxidative stress. Oveson *et al*. [59] found that TUDCA protected mice photoreceptors from oxidative stress damage by reducing the accumulation of superoxide radicals in the outer retinas after light exposure (measured with hydroethidine). Tao *et al*. [66] demonstrated that TUDCA could alter the retinal oxidation status in MNU-induced RD by reducing the level of MDA while increasing the content of SOD, the former was a stable metabolite of lipid peroxidation (measured with total bile acids colorimetric assay) and the latter was an endogenous antioxidant (measured with the SOD Assay Kit-WST). Mantopoulos *et al*. [72] revealed that TUDCA completely reversed the increased concentration of protein carbonyl after RDT for 24 hours (measured with ELISA), which illustrated that TUDCA reduced protein oxidation through ROS.

#### 3.3.7 The Effect of TUDCA on Vascular Abnormalities

Four studies provided the evaluation of retinal vasculature after treatment with TUDCA. Yang *et al*. [67] found that after the onset of diabetes, the retinas of rats exhibited focal dilation of retinal capillaries, with vessels tortuosity and sac-like bulges in the vessel wall. However, TUDCA alleviated vascular abnormalities in the retinas of diabetic rats (measured with the infusion of FITC-Dextran). In addition, It was also decreased leukocyte adhesion in the retinas of diabetic rats. Wang *et al*. [54] revealed that the protective effect of TUDCA on retinal vascular abnormalities in DR was associated with inhibiting the expression of VEGF (measured with IHC). Woo *et al*. [71] showed that systemically treated with TUDCA before and after laser-induced CNV, was able to reduce CNV fluorescein leakage (measured with Fluorescein fundus angiography (FFA)) and lesion size, improve vascularity (measured with HE staining), which was associated with suppression of early VEGF elevation in the retinas after laser injury (measured with ELISA). Fernández-Sánchez *et al*. [64] revealed that TUDCA was able to protect vascular degeneration, and treated animals displayed better-preserved capillary networks including more vessels and more capillary loops compared with untreated P23H rats which were observed with a reduction of capillary contents in the deep capillary plexus and evident loss of capillary loops (measured with histochemistry).

#### 3.3.8 Other Effects

Besides its neuroprotective effect on photoreceptor cells, TUDCA also protects from vascular and glial degeneration. Fernández-Sánchez *et al*. [64] found that TUDCA treatment increased the number of astrocytes and reduced the presence of Müller cells process clusters in the outer retina of P23H rats (measured with IHC). Therefore, the ability of TUDCA to protect retinal tissue from degeneration was powerful, which included affecting multiple retinal cells and preventing degeneration even at the late stages of the disease. Moreover, TUDCA altered body weight and blood glucose in different models. Phillips *et al*. [57] found that TUDCA-treated rd10 mice and wild-type C57BL/6J showed decreased body weight over the treatment period. The same phenomenon was seen in experiments targeting bbs1 mice [60]. However, it did not significantly alter weight in diabetes mellitus (DM) mice, but early treatment lowered blood glucose levels in DM animals [68].

## 4 DISCUSSION

### 4.1 Summary of Evidence

**Fig. (3):**
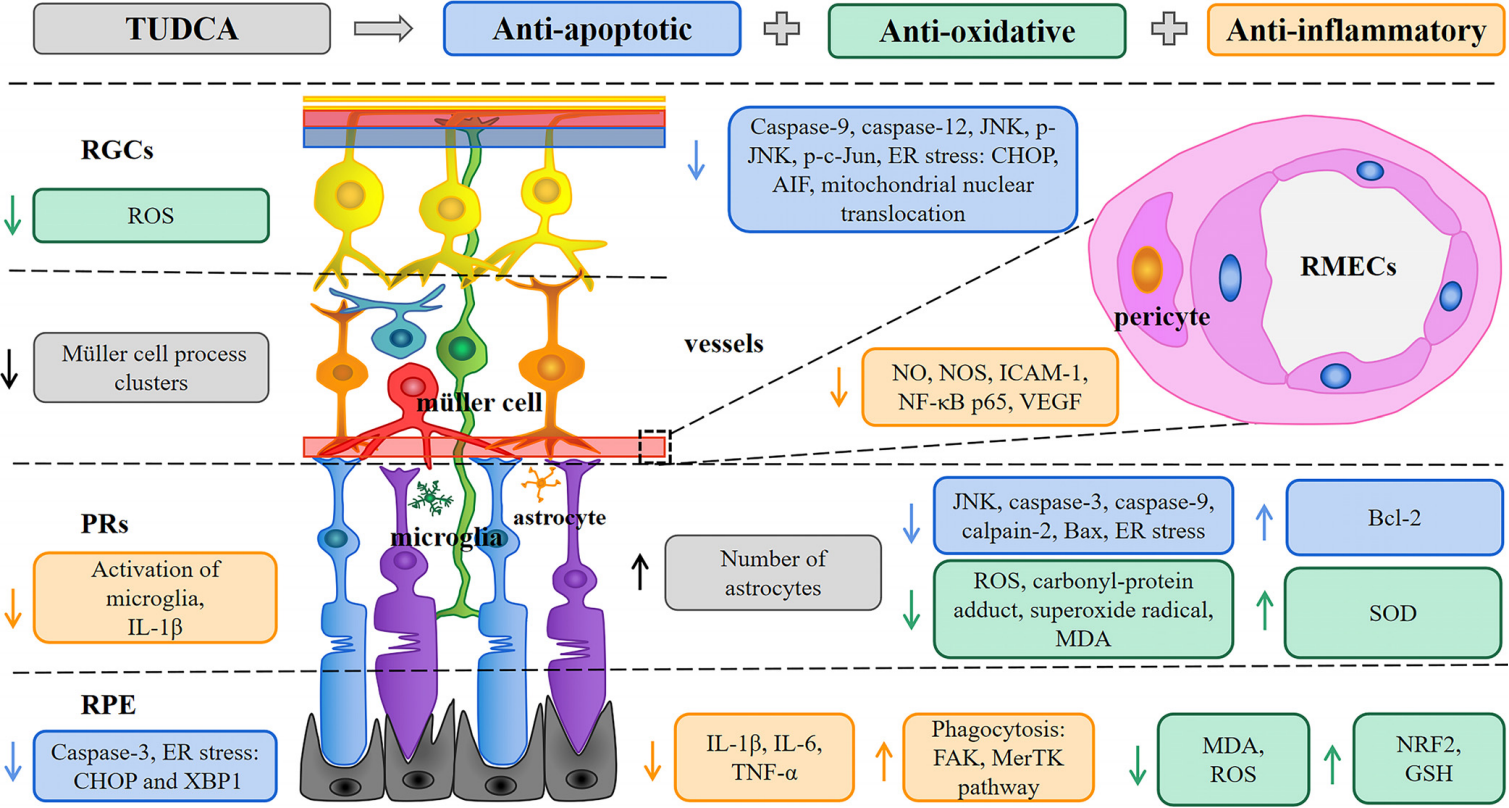
Mechanisms involved in TUDCA neuroprotective effects in retinal disorders. Anti-apoptotic (blue), anti-oxidant (green), anti-inflammatory (orange), others (grey) of TUDCA described in retinal disease models, at the level of RGCs, photoreceptors (PRs), RPE, and vessels. The upward arrow indicates the promoting effect of TUDCA, and the downward arrow indicates its inhibiting effect.

There is a great need for neuroprotective therapies for retinal disease since it is conducive to improving the life quality of patients and alleviating social problems caused by vision loss and eventual blindness [74-76]. Ideal neuroprotective strategies include increasing the survival of neurons by preserving neuronal structure and function [4]. Our study summarized the retinal protective effect of TUDCA, a compound found naturally in the bile acid of hibernating bears, which was described in the literature as a neuroprotective agent capable of ameliorating the damage of RP, RD, DR, CNV, RDT, LHON, and abnormity of RGCs and RPEs. Although the categories of diseases are very broad, all the above-mentioned retinal disorders involve similar pathological changes, that are, the structural and functional abnormalities of nerve cells or other cell components of the retina that constitute primary to tertiary neurons [42]. Importantly, the evaluation results of this systematic review demonstrated that TUDCA often targets these final mechanisms of varies diseases, and plays a therapeutic effect eventually. The neuroprotective effect of it involved a variety of mechanisms that are related to inhibiting apoptosis, decreasing inflammation, attenuating oxidative stress, suppressing endoplasmic reticulum stress, and reducing angiogenesis (Fig. **3**). These results provide sufficient evidence for further clinical studies.

### 4.2 Limitations

Some limitations of this systematic review should be noticed. 1) Studies evaluated in this systematic review were inconsistent in important aspects, such as the models of retinal diseases, duration and dose of TUDCA therapy, and routes of administration which reinforced the heterogeneity of the studies. Based on the methodological heterogeneity among studies, grouping statistics were not considered. Thus, meta-analyses were not performed for the accessed data. 2) This systematic review was limited to studies published in English only, so there are language and regional differences, and literature published in other languages may be missed, which may affect the results and extrapolation of the systematic review. 3) Only *in vivo* studies that used rodent models were included, which may have restricted the analysis of some studies. 4) The sample size varied greatly among the studies. The largest study had 40 mice per group, while some experiments had as little as 4 animals per group. For ethical and economic considerations, it is crucial to design animal experiments well to ensure that the data obtained are reliable, especially in terms of sample size [77]. 5) The SYRCLE’s assessment showed that the overall quality of the included studies was not high. It is suggested that attention should be paid to the detailed reporting of random sequence generation, allocation concealment, random outcome assessment, and the use of blinding in the future, which will improve the reliability and rigor of the studies.

### 4.3 Future Directions

For multiple neurodegenerative diseases, TUDCA therapy has been translated to clinical practice [78]. At present, there are 34 TUDCA trials listed in clinical trials.gov (https://www.clinicaltrials.gov/, accessed October 15, 2022), 12 of them focused on neurodegenerative diseases. No clinical study of TUDCA for retinal diseases has been registered, but a study to evaluate UDCA as an adjuvant treatment for rhegmatogenous RDT is retrieved (NCT02841306; University of Lausanne, France) [4]. Study details: a single dose of UDCA is administered prior to surgery to repair the detachment, and 3 months after surgery, the patients continued to receive UDCA treatment to promote the survival of photoreceptors. The results are not yet available. Three trials apply TUDCA in diabetic patients. There is an opportunity to utilize these trials to evaluate the visual outcomes of diabetic patients who may develop or already have DR [4]. Therefore, as one of the attractive candidates, rapidly translating TUDCA into the clinic is a goal in the future.

The most frequently used method of administration in animal experiments was subcutaneous or intraperitoneal injection (common dose: 500 mg/kg). As a retinal neuroprotection strategy, ideal delivery would target the eye rather than systemically, with the goal of delivering the optimal therapeutic dose to the retina while minimizing side effects. At present, the majority of therapies for diseases of the posterior segment require intraocular injections in order to effectively deliver the active agents to the target site. However, this method of administration is invasive and carries a risk of complications [79]. Therefore, one of the keys for further research will aim at developing a feasible drug delivery method to achieve therapeutic levels in the form of topical treatment. Intraocular drug delivery systems are emerging therapeutic tools capable of releasing the loaded molecules overtime and avoiding repeated intravitreal injections [80, 81]. Alicia Arranz-Romera has reported a multi-loaded intraocular drug delivery system with two different neuroprotectant agents, glial cell-line-derived neurotrophic factor (GDNF) and TUDCA, combined into a single microcarrier. In this system, the payload rates of the two drugs were 48.86±1.49% (GDNF) and 78.58±10.40% (TUDCA) respectively, and these optimized microparticles were continuously released *in vitro* over 91 days [82]. Sanchez [62] successfully delivered TUDCA into the vitreous cavity of rats with this type of microsphere, and achieved sustained delivery. In the model of rats with P23H photoreceptor degeneration, they showed modest functional preservation when the microspheres were injected intravitreally once a month, compared with those systemic injections weekly. Kitamura [70] tried to deliver TUDCA to the retina in the form of eye drops, and it partially preserved the optic nerve, but eye drops delivery to the posterior segment has its own special challenges, such as the obstruction of blood vessels, conjunctiva, sclera, and choroid tissues, as well as blood-retinal barriers. Therefore, optimizing the delivery methods to provide more effective levels of sustained delivery may be the next key research direction for TUDCA in the treatment of eye diseases.

## CONCLUSION

In this systematic review, TUDCA, as a neuroprotective agent used in retinal disorders, exhibited anti-apoptosis, anti-inflammatory, anti-oxidant, anti-ER stress, and anti-angiogenesis properties, and had a neuroprotective effect on *in vivo* and *in vitro* models of retinal disorders. Those results reinforced the currently available evidence that TUDCA could be a promising therapeutic agent in retinal disease treatment. However, well-designed randomized controlled clinical trials are necessary to appraise the efficacy of TUDCA in the clinical setting.

## Figures and Tables

**Fig. (2):**
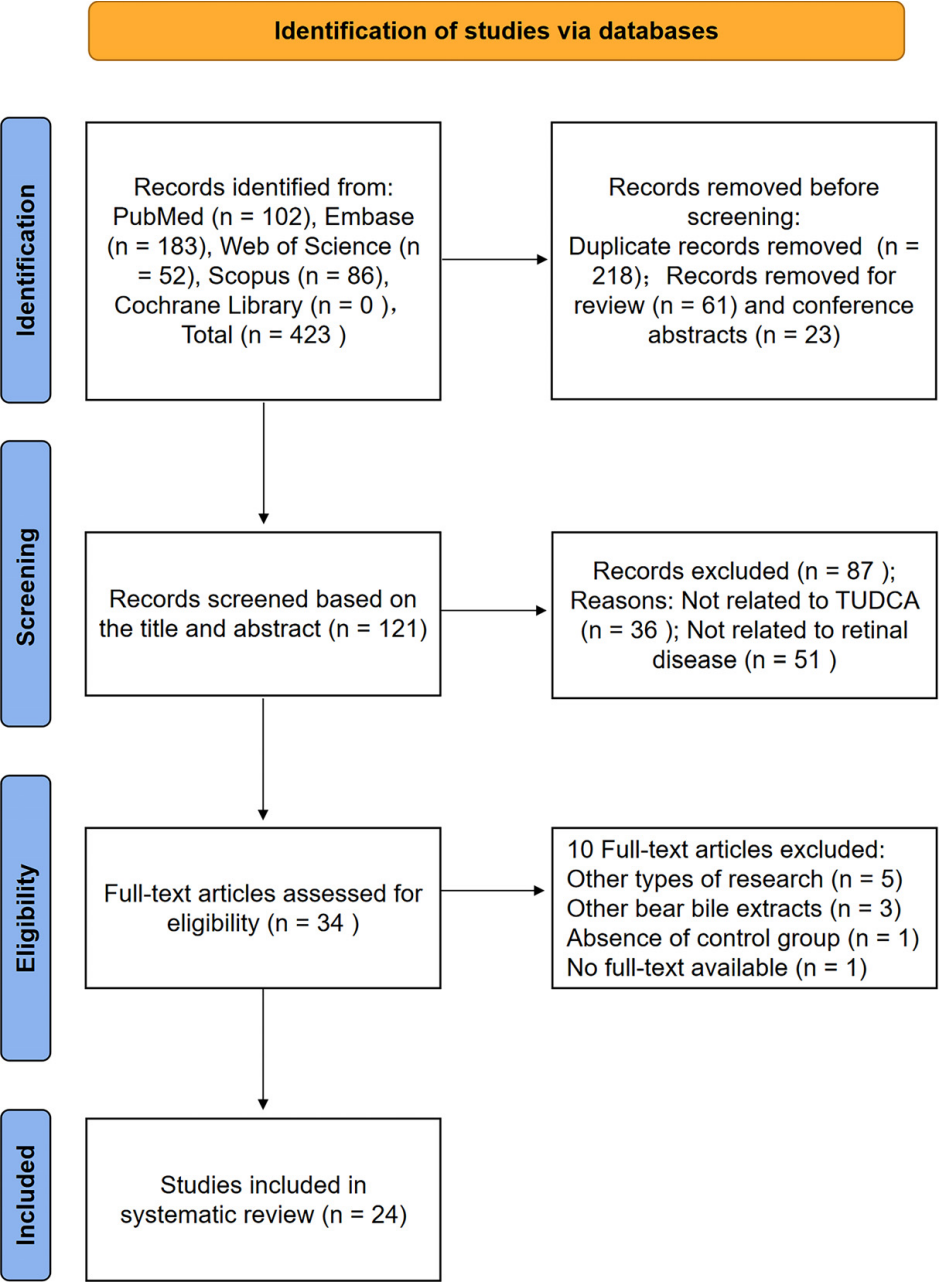
Flow chart of the results according to the search strategies.

**Table 1:** *In vitro* studies on TUDCA effect in cells and tissues of retinal disorder.

**Author, Year (Country)**	**Theme**	**Cells or Tissues**	**Insult**	**TUDCA ** **Concentration ** **(Incubation Time)**	**Laboratory ** **Techniques**	**Major Findings**	**Mechanisms**
Guzel Bikbova, 2016 (Japan) [50]	RD	RE from SD rats (Japan SLC Co., Ltd)	100 µg/ml glucose-AGE-BS for 7 days	100 µM TUDCA for 7 days	TUNEL staining, IHC	Promotion of regenerating neurites and reduction of apoptosis by reducing JNK and caspase-9	Anti-apoptosis, anti-endoplasmic reticulum (ER) stress
Alejandra Daruich, 2022 (France, USA) [51]	RD	1) WERI-Rb-1 human cone cell line (ATCC, USA); 2) RE from Wistar rats (Janvier Labs, France)	1) WERI-Rb-1: 20 mg/mL albumin for 24 h; 2) RE:12 mg/mL albumin for 6 h	1) WERI-Rb-1:1 µM UDCA for 24 h; 2) RE: 10 ng/mL TUDCA for 6 h	IHC, WB, TUNEL staining, RNA sequencing	Protection of cells from albumin toxicity by reducing apoptosis, necroptosis, and microglia activation	Anti-apoptosis, anti-ER stress
J.M. Gaspar, 2013 (Portugal) [52]	DR	Retinal neural cells from Wistar rats	25 mM D-glucose for 7 days	100 µM TUDCA every other day for 7 days	Annexin-V fluorescein isothiocyanate (FITC) staining, TUNEL staining, WB, ICC, H_2_DCFDA, dot blot	Decrease cell death by preventing the release and mito-nuclear translocation of AIF and down-regulating biomarkers of oxidative stress, protein carbonyl groups, and ROS production	Anti-apoptosis, anti-oxidative
Toshiyuki Oshitari, 2014 (Japan) [53]	DR	RE from STZ (55 mg/kg) induced diabetic SD rats. (Japan SLC Co., Ltd)	45 mM high-glucose for 7 days	100 µM TUDCA for 7 days	TUNEL staining, IHC, WB	The neuroprotective effect of TUDCA is correlated with the suppression of p-c-Jun and p-JNK expression.	Anti-apoptosis, anti-ER stress
Chunfei Wang, 2016 (China) [54]	DR	HRMECs (Guangzhou Jennio Biotech Co., Ltd, China)	4.5 g/L glucose for 24 h, 48 h, and 72 h	0.2 μM, 1.0 μM, 5.0 μM, 25 μM and 125 μM TUDCA for 24 h, 48 h and 72 h, respectively	MTT assay, ELISA, WB	Inhibition of inflammation by decreasing ICAM-1, NOS, NF-κB P65, VEGF	Anti-apoptosis, anti-inflammation, anti- neovascularization
Hiromi Murase, 2015 (Japan) [55]	RPE damage	1) ARPE-19 line (American Type Culture Collection, USA); 2) hRPE cells (Lonza, USA); 3) POS from Porcine Eyes	100 µM H_2_O_2_ for 6 h	1) ARPE-19 cells: 30 to 300 μM for 2 h; 2) hRPE cells: 300 μM for 2 h	Fluorescence labeling, CM-H_2_DCFDA, WB	Enhancement of phagocytosis in POS and reversion of phagocytosis impairment in RPE cells by activating MerTK pathway	Activate of MerTK pathway
Reem Hasaballah Alhasani, 2020 (China, UK, Saudi Arabia) [56]	RPE damage	ARPE-19 cells (ATCC, USA)	1) Oxidative damage: 750 µM H_2_O_2_ for 24 h; 2) ER stress: 1 µM thapsigargin for 24 h	100 µM TUDCA for 24 h	MTT assay, TUNEL staining, 6-Carboxy-H_2_DCFDA, qRT-PCR, CASPASE-Glo 3/7 assay, ELISA	1) Increase of cell viability and decrease apoptosis by inhibiting caspase-3,7; 2) suppression of ER stress by down regulating CHOP and XBP1; 3) Improvement of antioxidant capacity by reducing ROS and MDA, upregulating antioxidant genes, GSH and NRF2; 4) Alleviation inflammation by decreasing IL-1β, IL-6, and TNF-α	Anti-apoptosis, anti-ER stress, anti-oxidative, anti-inflammation

**Table 2:** *In vivo* studies on TUDCA effect in animals of retinal disorder.

**Author, ** **Year ** **(Country)**	**Theme**	**Animals** ** (Sex and Old)**	**Models**	**Sample ** **Size(n)**	**TUDCA Route and Dose**	**Frequency and Duration**	**Follow ** **Up**	**Tissues**	**Laboratory Techniques**	**Major Findings**
M.Joe Phillips, 2008 (USA, Korea) [57]	RP	Rd10 mice (sex not specified, neonatal)	Rd10 mutant mice	*n* = 17 or *n* = 14 per group	Subcutaneous injections (500 mg/kg body weight)	Once every 3 days, P6-P30, 8 times total	P30	Eye ball	ERG, immunolabeling, TUNEL staining	Preserve rod and cone function and overall photoreceptor numbers, reduction of apoptosis in RP model
Laura Fernández-Sánchez, 2011 (Spain) [58]	RP	P23H rats (sex not specified, neonatal)	P23H mutant rats	*n* = 18-22 per group	Intraperitoneal injections (500 mg/kg body weight)	Once a week, P21-P120	P120	Eye ball	ERG, IHC, TUNEL staining	Preserve cone and rod structure and function, together with their contacts with their postsynaptic neurons, reduction of apoptosis in the RP model
Brian C. Oveson, 2011 (USA) [59]	RP	1) Rd10 mice (both sexes, neonatal); 2) BALB/c mice (female, 4-6 weeks old)	1) Rd10 mutant mice; 2) BALB/c mice (5000 lux for 8 h)	*n* = 8 per group	Subcutaneous injections (500 mg/kg body weight)	1) Rd10: once every 3 days, P6 to P30 or P50; 2) BALB/c: one day and again 1 h prior to light exposure, single	1) Rd10: P30 or P50; 2) BALB/c: Day 1 or Day 7	The eyeball and whole retina	ERG, Fluorescence labeling, retinal flat-mount, HE staining	Preserve photoreceptor structure and function by anti-oxidative in RP model
Arlene V.Drack, 2012 (USA) [60]	RP	1) Bbs1 mice; 2) Rd10 mice (sex not specified, neonatal)	1) Bbs1 mutant mice; 2) Rd10 mutant mice	*n* = 4-6 per group	Subcutaneous injections (500 mg/kg body weight)	1) Bbs1: twice a week, P40-P120; 2) Rd10: once every 3 days, P6-P38	1) Bbs1: P120; 2) Rd10: P30 or P38	Eye ball	ERG, OCT, HE staining	1) Preserve of ERG b-waves, the outer nuclear layer(ONL), and prevention of obesity in Bardet-Biedl syndrome mode; 2) Preserved ERG b-waves and the ONL in RP model
Agustina Noailles, 2014 (Spain) [61]	RP	P23H rats (sex not specified, neonatal)	P23H mutant rats	*n* = 6 or *n* = 4 per group	Intraperitoneal injections (500 mg/kg body weight)	Once a week, P20-P120	P120	The eyeball and whole retina	IHC	Reduction of microglial cell number, prevention of activation of microglial cells and inhibition of the number and activation of macrophages, by anti-inflammatory effect in RP model
Laura Fernández-Sánchez, 2017 (Spain) [62]	RP	P23H rats (sex not specified, neonatal)	P23H mutant rats	*n* = 17 per group	Intravitreal injections of TUDCA-loaded PLGA MSs (5.05 ± 0.11 μg TUDCA/mg MSs)	Once a month, P30-P90 or P120	P30, P60, P90 and P120	Eye ball	ERG, immunofluorescence, HE staining	Preserve photoreceptor function and preserve the synaptic contacts of photoreceptors with bipolar and horizontal cells in the RP model
Xun Zhang, 2019 (Scotland) [63]	RP	Rpgr knockout mice (both sexes, neonatal)	Rpgr knockout mice	*n* = 6 per group	Intraperitoneal injections (500 mg/kg body weight)	Once a week, P30-P120	P120	Eye ball	HE staining, immunofluorescence, qRT-PCR, WB, TUNEL staining	Prevention of photoreceptor degeneration and reduction of apoptosis in RP by suppressing microglial activation and inflammation
Laura Fernández-Sánchez, 2022 (Spain) [64]	RP	P23H rats (sex not specified, neonatal)	P23H mutant rats	*n* = 12 or *n* = 15 or *n* = 11 per group	Intraperitoneal injections (500 mg/kg body weight)	Once a week, P21-P120	P120	The eyeball and whole retina	Retinal flat-mount, IHC, nicotinamide adenine dinucleotide phosphate-diaphorase (NADPH-d) histochemistry	Neuroprotective effect on photoreceptor cells and protection of vascular and glial degeneration in RP model
Eric C. Lawson, 2016 (USA) [65]	RD	Rd1 mice (sex not specified, neonatal)	Rd1 mutant mice	*n* = 6 per group	Intraperitoneal injections (500 mg/kg body weight)	Once a day, P6-P21	P21	Eye ball	ERG, toluidine blue staining	Preserve function and structure in a model of rapid RD
Ye Tao, 2019 (China) [66]	RD	C57BL/6 mice (both sexes, 8 weeks old)	MNU(60 mg/kg body weight single, ip)	*n* = 40 per group	Subcutaneous injections (500 mg/kg body weight)	Daily for consecutive 7 days for 3 days before and 4 days after MNU	Day 7 after MNU	The eyeball and whole retina	Optokinetic behavioral test, ERG, retinal flat-mount, SD-OC-OCT, HE staining, immunofluorescence, IHC, TUNEL staining, qRT-PCR, Multi-electrode array (MEA)	Protection of visual function(visual acuity, contrast sensitivity, optokinetic performance) and structure by downregulating the levels of apoptosis-related factors Caspase-3, calpain-2, and Bax. Amelioration of the retinal oxidation status by decreasing MDA levels and increasing superoxide dismutase (SOD) contents in the RD model
Liping Yang, 2013 (China, USA) [67]	DR	SD rats (female, 8 weeks old, 180g)	STZ(60 mg/kg body weight, single, ip)	*n* = 18 per group	Intraperitoneal injections (500 mg/kg body weight)	Once a day for 8 weeks, beginning 5 days after STZ	2, 4, 6, and 8 weeks	The eyeball and whole retina	Retinal flat-mount, immunofluorescence, perfusion labeling, fundus fluorescein angiograph, HE staining, IHC, Real-time PCR, WB	1) Protection of RGC death in diabetic retinas by inhibiting caspase-12 and p-c-Jun 1 levels; 2) Prevention of neuronal loss and vascular abnormalities by reducing CHOP expression; 3) Prevention of DR by inhibiting the activation of ER stress pathways
Chunfei Wang, 2016 (China) [54]	DR	SD rats (male, age not specified, 180-200g)	High glucose fat diet + STZ (30 mg/kg body weight, single, ip)	*n* = 10 per group	Intraperitoneal injections (750, 500, 250 mg/kg body weight)	Once a day for 2 months	2 months	Eye ball	ELISA, HE staining, IHC	Amelioration of DR by decreasing NO content and down-regulating the inflammatory factors expression of ICAM-1, NOS, NF-κB p65, and VEGF
Jieming Fu, 2021 (USA) [68]	DR	C57BL/6J (both sexes, 8-10 weeks old)	STZ (50 mg/kg body weight, single, IP)	*n* = 121 total	Intraperitoneal injections (500 mg/kg body weight)	Twice a week for 10 weeks, starting 1 or 3 weeks after induction of diabetes	Starting from 4 weeks after STZ, every two weeks or every four weeks	/	Optomotor Response(OMR), ERG	Preserve visual function in type I diabetes model by ameliorating ERG a-wave, b-wave, and OP2 deficits and maintaining spatial frequency and contrast sensitivity threshold
Violeta Gómez-Vicente, 2015 (Spain) [69]	RGCs injury	SD rats (sex not specified, 12-16 weeks old)	Intravitreal injection of NMDA (3 μl of 20 mM, single)	*n* = 5-6 per group	Intraperitoneal injections (500 mg/kg body weight)	Once a day for 6 days	Day 4, day 7 after TUDCA	The eyeball and whole retina	ERG, IHC, retinal flat mount	Protection of retinal function by increasing RGC density
Yuta Kitamura, 2019 (Japan) [70]	RGCs injury	SD rats (male, 7-10 weeks old)	Optic nerve crush, single	*n* = 6 per group	Topical administration (100 mM, one drop)	Once every 12 hours for 14 days	2 weeks	The eyeball and whole retina	IHC, TUNEL staining	Protection of the densities of RGCs and prevention of axons damage in optic nerve
Se Joon Woo, 2010 (Korea) [71]	CNV	BN rats (male, adult)	512 nm Argon laser photocoagulation, single (spot size of 100 mm, at 150 mW, over 100 ms)	*n* = 13 per group	Intraperitoneal injections (100 mg/kg body weight)	Once a day, from 1 day before to 14 days after the laser	14 days after CNV induction	Eye ball	Fluorescein fundus angiography (FFA), HE staining, ELISA	Reduction of CNV fluorescein leakage and lesion size, improvement of vascularity, by suppressing early VEGF elevation in the retina after laser injury, which might be associated with anti-inflammatory action
Dimosthenis Mantopoulos, 2011 (USA) [72]	RDT	BN rats (male, adult, 200-250 g)	Transscleral subretinal injection of 50 mml of 1% sodium hyaluronate, single	*n* = 6 per group	Intraperitoneal injections (500 mg/kg body weight)	Once a day, first injection 24 hours prior to the induction of RD, the treatment was started 6 hours after RD, for 3day or 5day	3 and 5 days after RDT induction	Eye ball	TUNEL staining, IHC, ELISA, caspase 2, 3, 8, 9 colorimetric assay, WB	1) Preserve of photoreceptors after RDT, reduction of apoptosis, which might be associated with suppression of caspases 3 and 9, as well as carbonyl-protein adducts; 2) Reduction of protein oxidation through ROS
Tao Zhang, 2012 (USA) [73]	LHON	Lrat-/- mice (sex not specified, neonatal)	Lrat-/- mutant mice	*n* = 6-8 per group	Subcutaneous injections (500 mg/kg body weight)	Once every 3 days, starting at P9 to P28	P28	The eyeball and whole retina	Retinal flat mount, IHC, WB	1) Reduction of ER stress and apoptosis in Leber’s model; 2) Postponement of cone degeneration and promotion of the degradation of cone membrane-associated proteins by enhancing the ER-associated protein degradation pathway

**Table 3:** Bias risk of the *in vivo* studies.

Study/Bias	Selection Bias			Performance Bias		Detection Bias		Attrition Bias	Reporting Bias	Other Bias	Quality Score (“YES” Items)
	Sequence Generation	Baseline Characteristics	Allocation Concealment	Random Housing	Blinding	Random Outcome Assessment	Blinding	Incomplete Outcome Data	Selective Outcome Reporting	Other Sources of Bias	
M.Joe Phillips, 2008 (USA, Korea)	NC	YES	NC	NC	NC	NC	YES	YES	YES	YES	5
Laura Fernández-Sánchez, 2011 (Spain)	NC	YES	NC	YES	YES	NC	YES	YES	YES	YES	7
Brian C. Oveson, 2011 (USA)	NC	YES	NC	NC	YES	NC	YES	YES	YES	YES	6
Arlene V. Drack, 2012 (USA)	NC	YES	NC	NC	NC	NC	YES	YES	YES	YES	5
Agustina Noailles, 2014 (Spain)	NC	YES	NC	YES	YES	NC	YES	YES	YES	YES	7
Laura Fernández-Sánchez, 2017 (Spain)	NC	YES	NC	YES	YES	NC	YES	YES	YES	YES	7
Xun Zhang, 2019 (Scotland)	NC	YES	NC	NC	NC	NC	YES	YES	YES	YES	5
Laura Fernández-Sánchez, 2022 (Spain)	NC	YES	NC	YES	YES	NC	YES	YES	YES	YES	7
Eric C. Lawson, 2016 (USA)	NC	YES	NC	NC	NC	NC	YES	YES	YES	YES	5
Ye Tao, 2019 (China)	NC	YES	NC	YES	YES	NC	YES	YES	YES	YES	7
Liping Yang, 2013 (China, USA)	NC	YES	NC	NC	NC	NC	YES	YES	YES	YES	5
Chunfei Wang, 2016 (China)	NC	YES	NC	YES	YES	NC	YES	YES	YES	YES	7
Jieming Fu, 2021 (USA)	NC	YES	NC	NC	NC	NC	YES	YES	YES	YES	5
Violeta Gómez-Vicente 2015 (Spain)	NC	YES	NC	NC	NC	NC	YES	YES	YES	YES	5
Yuta Kitamura, 2019 (Japan)	NC	YES	NC	NC	NC	NC	YES	YES	YES	YES	5
Se Joon Woo, 2010 (Korea)	NC	YES	NC	NC	NC	NC	YES	YES	YES	YES	5
Dimosthenis Mantopoulos, 2011 (USA)	NC	YES	NC	NC	NC	NC	YES	YES	YES	YES	5
Tao Zhang, 2012 (USA)	NC	YES	NC	NC	NC	NC	YES	YES	YES	YES	5

## LIST OF ABBREVIATIONS

AD: Alzheimer Disease
AIF: Apoptosis-inducing Factor
AMD: Age-related Macular Degeneration
ARPE-19: Adult Retinal Pigment Epithelial Cell Line-19
Bcl2: B-cell Lymphoma 2
BN: Brown Norway
CDCA: Chenodeoxycholic Acid
CHOP: CCAAT-enhancer-binding Protein Homologous Protein
CNV: Choroidal Neovascularization
H_2_DCFDA: 2',7'-Dichlorodihydrofluorescein Diacetate
DM: Diabetes Mellitus
DR: Diabetic Retinopathy
ELISA: Enzyme-linked Immunosorbent Assay
ER: Endoplasmic Reticulum
ERG: Electroretinogram
FAK: Focal Adhesion Kinase
FFA: Fluorescein Fundus Angiography
FITC: Fluorescein Isothiocyanate
GCL: Ganglion Cell Layer
GDNF: Glial Cell-line-derived Neurotrophic Factor
Glucose-AGE-BS: Glucose-advanced Glycation End Products-bovine Serum Albumin
GSH: Glutathione
H_2_O_2_: Hydrogen Peroxide
HD: Huntington’s Disease
HE: Hematoxylin-eosin
HRMECs: Human Retinal Microvascular Endothelial Cells
hRPE: Human Retinal Pigment Epithelium
ICAM-1: Intercellular Adhesion Molecule-1
ICC: Immunocytochemistry
IHC: Immunohistochemistry
IL-1β: Interleukin-1β
IL-6: Interleukin-6
INL: Inner Nuclear Layer
IS: Inner Segment
JNK: c-Jun N-terminal Kinase
LHON: Leber’s Hereditary Optic Neuropathy
MDA: Malondialdehyde
MEA: Multi Electrode Array
MerTK: Mer Tyrosine Kinase Receptor
MeSH: Medical Subject Headings
MNU: N-methyl-N-nitrosourea
NADPH-d: Nicotinamide Adenine Dinucleotide Phosphate-diaphorase
NF-κB: Nuclear Factor Kappa-B
NMDA: N-methyl-D-aspartate
NOS: Nitric Oxide Synthase
NRF2: Nuclear Factor Erythroid 2-related Factor 2
OCT: Optical Coherence Tomography
OMR: Optomotor Response
ONL: Outer Nuclear Layer
OP2: Oscillatory Potential 2
OPL: Outer Plexiform Layer
OS: Outer Segment
p-c-Jun: Phosphorylated c-Jun
p-JNK: Phosphorylated JNK
P23H: Pro 23 His
PD: Parkinson Disease
PLGA MSs: Poly D, L-lactic-co-glycolic Acid Microspheres
PNA: Peanut Agglutinin
POS: Photoreceptor Outer Segment
PRISMA: The Preferred Reporting Items for Systematic Reviews and Meta-Analyses
PRISMA-CHM: PRISMA Extension for Chinese Herbal Medicines
PRs: Photoreceptors
qRT-PCR: Real-time Quantitative Polymerase Chain Reaction
RD: Retinal Degeneration
RDT: Retinal Detachment
RE: Retinal Explants
RGC: Retinal Ganglion Cell
RGCs: Retinal Ganglion Cells
ROS: Reactive Oxygen Species
RP: Retinitis Pigmentosa
RPE: Retinal Pigment Epithelium
SD: Sprague-dawley
SOD: Superoxide Dismutase
STZ: Streptozotocin
SYRCLE: Systematic Review Centre for Laboratory Animal Experimentation
TCM: Traditional Chinese Medicine
TNF-α: Tumor Necrosis Factor-α
TUDCA: Tauroursodeoxycholic Acid
TUNEL: Terminal-deoxynucleotidyl Transferase-mediated Nick End Labeling
UDCA: Ursodeoxycholic Acid
VEGF: Vascular Endothelial Growth Factor
WB: Western Blot
XBP1: X-box Binding Protein-1

## STANDARDS OF REPORTING

PRISMA guidelines and methodology were followed.

## SUPPLEMENTARY MATERIAL

PRISMA checklist is available as supplementary material on the publisher’s website along with the published article.
